# Clinicopathological features and surgical outcomes of neuroendocrine tumors of ampulla of Vater

**DOI:** 10.1186/s12876-017-0630-9

**Published:** 2017-05-31

**Authors:** Kwangho Yang, Sung Pil Yun, Suk Kim, Nari Shin, Do Youn Park, Hyung Il Seo

**Affiliations:** 1 Department of Surgery, Division of Hepato-Biliary-Pancreatic Surgery and Transplantation, Pusan National University Yangsan Hospital, 20, Geumo-ro, Mulgeum-eup, Yangsan, Gyeongsangnam-do 50612 South Korea; 2 Research Institute for Convergence of Biomedical Science and Technology, Pusan National University Yangsan Hospital, 20, Geumo-ro, Mulgeum-eup, Yangsan, Gyeongsangnam-do 50612 South Korea; 3 Department of Surgery, Biomedical Research Institute, Pusan National University Hospital, 179, Gudeok-Ro, Seo-Gu, Busan, 602-739 South Korea; 4 Department of Radiology, Biomedical Research Institute, Pusan National University Hospital, 179, Gudeok-Ro, Seo-Gu, Busan, 602-739 South Korea; 5 Department of Pathology, Pusan National University Yangsan Hospital, 20, Geumo-ro, Mulgeum-eup, Yangsan, Gyeongsangnam-do 50612 South Korea; 6 Department of Pathology, Biomedical Research Institute, Pusan National University Hospital, 179, Gudeok-Ro, Seo-Gu, Busan, 602-739 South Korea

**Keywords:** Neoplasms, Neuroendocrine tumors, Ampulla of Vater, Pancreaticoduodenectomy, Treatment outcome

## Abstract

**Background:**

The study aims to investigate the clinicopathological features and surgical outcomes of neuroendocrine tumors of ampulla of Vater (NETAoVs) patients who underwent pancreaticoduodenectomy.

**Methods:**

From January 2007 to December 2014, 45 patients underwent pancreaticoduodenectomy for malignant disease of the ampulla of Vater in our institution. Of those, 5 patients were diagnosed as neuroendocrine tumors. The data included age, sex, presenting symptoms, preoperative imaging, preoperative type of biopsy results, type of operation, pathologic findings and survival status.

**Results:**

The patient’s mean age was 55.2 ± 9.7 years. Endoscopic ultrasound guided biopsy was performed in 4 patients and gastroduodenoscopic biopsy was performed in one patient. All showed neuroendocrine tumor without mitosis. Mean tumor size was 1.9 ± 0.56 cm (range, 1.2–2.0 cm). Lymph node metastases were detected in two patients. All patients were synaptophysin-positive. Median periods of follow-up were 45 months (range, 43–78 months). Recurrence after operation occurred in two patients. 4 patients were alive at the last follow-up.

**Conclusions:**

Radical resection for NETAoVs can provide the information of status of lymph node metastasis after surgery. However, correlation between lymph node metastasis and overall survival is uncertain to date.

## Background

Neuroendocrine tumors of ampulla of Vater (NETAoVs) are uncommon. To date, only approximately 120 NETAoVs have been described in the literature, most in less 10 cases reports [1–5]. The incidence and prevalence of neuroendocrine tumor seems to have increased in recent years, most likely due to diagnostic technical improvements and endoscopic healthcare surveillance [6].

Computed tomography (CT), magnetic resonance imaging (MRI) and endoscopic ultrasound (EUS) guided biopsy are the main tools for preoperative examinations, but immunohistochemical staining assessment using biopsied specimen is important for diagnosis. There is no standard treatment for NETAoVs, because their natural history and prognostic factors remain unclear. In spite of long term survival has been reported after local excision, many surgeons favor pancreaticoduodenectomy due to the high incidence of lymph node metastasis [6, 7]. In this study, we report clinicopathological features and surgical outcomes of 5 NETAoVs patients who underwent pancreaticoduodenectomy.

## Methods

From January 2007 to December 2014, 45 patients underwent pancreaticoduodenectomy for malignant disease of the ampulla of Vater in our institution. The surgeries were performed by the same operator. Of those, 5 patients were diagnosed as NETAoVs. The data included age, sex, presenting symptoms, preoperative imaging, preoperative type of biopsy results, type of operation, pathologic findings and survival status. CT and MRI were performed to assess the presence of locoregional lymph node metastases or distant metastases. The pathological data were assessed by the same pathologist, according to 2010 World Health Organization (WHO) classification, and 2006 European Neuroendocrine Tumour Society (ENETS) and the seventh edition International Union Against Cancer (UICC) staging systems [8–10] (Table 1). Immunohistochemical analysis included CD56, synaptophysin and chromogranin A expression, and the Ki67 index was assessed for histological grading. The study was reviewed and approved by the Pusan National University Hospital Institutional Review Board.

**Table 1:** Staging system for neuroendocrine tumor of the ampulla of Vater

WHO classification (2010)								
	Grade 1				<2 mitoses/10 HPF and <3% Ki–67			
	Grade 2				2–20 mitoses/10 HPF or 3–20% Ki–67			
	Grade 3				>20 mitoses/10 HPF or >20% Ki–67			
TNM staging system								
	ENETS (2006)				UICC (7th edition, 2009)			
*T – primary tumor*								
Tx	Primary tumour cannot be assessed							
T0	No evidence of primary tumour							
T1	Invasion of lamina propria or submucosa and size ≤ 1 cm				Limited to ampulla of Vater or sphincter of Oddi			
T2	Invasion of muscularis propria or size > 1 cm				Invasion of the duodenum wall			
T3	Invasion of the pancreas or retroperitoneum				Invasion of the pancreas			
T4	Invasion of the peritoneum or other organs				Invasion in peripancreatic soft tissues or other adjacent organs or structures			
*N – regional lymph nodes*								
Nx	Regional lymph nodes cannot be assessed							
N0	No regional lymph node metastasis							
N1	Regional lymph node metastasis present							
*M – distant metastasis*								
Mx	Distant metastasis cannot be assessed							
M0	No distant metastasis							
M1	Distant metastasis present							
Staging	Stage I	T1	N0	M0	Stage Ia	T1	N0	M0
	Stage IIa	T2	N0	M0	Stage Ib	T2	N0	M0
	Stage IIb	T3	N0	M0	Stage IIa	T3	N0	M0
	Stage IIIa	T4	N0	M0	Stage IIb	T1–3	N1	M0
	Stage IIIb	Any T	N1	M0	Stage III	T4	Any N	M0
	Stage IV	Any T	Any N	M1	Stage IV	Any T	Any N	M1

*ENETS* European Neuroendocrine Tumour Society staging system, *HPF* high power fields, *UICC* International Union Against Cancer staging system, *WHO* World Health Organisation classification

## Results

### Clinical findings and preoperative evaluation

The clinical features of the 5 patients are listed in Table 2. Mean age was 55.2 ± 9.7 years (range, 36–62 years) and the male to female ratio was 3:2. One patient presented with obstructive jaundice, which led to endoscopic retrograde cholangiopancreatography. In 4 patients, the tumor was discovered during gastroduodenoscopy as part of a regular medical check-up. No patient had specific neuroendocrine symptoms, and signs of Recklinghausen’s disease or Zollinger-Ellison syndrome [11, 12]. EUS-guided biopsy was performed in 4 patients and gastroduodenoscopic biopsy was performed in one patient. Histologically, all cases displayed neuroendocrine tumor without mitosis.

**Table 2:** Clinical features and outcomes of patients

Case	Sex	Age range	Symptom	L/N ratio	Biopsy result	Operation	Adjuvant CTx	Recurrence site	Treatment for recurrence	Survival outcome
1	Female	50–59	Incidental	62.1%	NET	PPPD	Yes	NED		78 months alive
2	Male	60–69	Jaundice	15.8%	NET	PPPD	Yes	Liver	RFA, CTx	67 months dead
3	Female	50–59	Incidental	40.9%	NET	PPPD	Yes	NED		45 months alive
4	Male	30–39	Incidental	52.7%	NET	PD	Yes	NED		44 months alive
5	Male	60–69	Incidental	23.4%	NET	PD	No	liver	CTx	43 months alive

*CTx* chemotherapy, *L/N ratio* lymphocyte/neutrophil ratio, *NED* no evidence of disease, *NET* neuroendocrine tumor, *PD* pancreaticoduodenectomy, *PPPD* pylorus preserving pancreaticoduodenectomy, *RFA* radiofrequency ablation

CT scan using a hepatopancreatic protocol was performed in all patients and MRI was performed in 3 patients. The imaging procedures showed an enhanced mural or intramural mass. Only one patient showed dilatation of main pancreatic duct in imaging study. Liver metastases were not detected in any patient. Positron emission tomography-CT performed in 4 patients showed fluorodeoxyglucose uptake in all the patients (mean SUVmax 4.2, range; 2.5–7).

### Treatment

Three patients underwent pylorus-preserving pancreaticoduodenectomy. The other two patients underwent conventional pancreaticoduodenectomy because severe adhesion between stomach and pancreas, and tumor invasion to duodenal first portion, respectively. Lymph node dissection was performed as standard extent for pancreas cancer. Duct-to-mucosa Pancreaticojejunostomy was performed in every case. There was no mortality. There were three minor complications (grade I or II according to Clavien-Dindo classification) including 2 delayed gastric emptying and a pancreas fistulae grade A. No patient was treated by radiological intervention or re-exploration. After surgery, adjuvant chemotherapy using etoposide-cisplatin intravenous administration with or without octreotide LAR intramuscular injection was done in 4 patients. Distant recurrence of NETAoVs occurred in 2 patients (case 2 and case 5). Case 2 suffered single liver metastasis at 10 months after surgery and underwent radiofrequency ablation for the lesion followed by chemotherapy with etoposide-cisplatin. However, multiple liver metastases still occurred and the patient expired at 67 months after surgery. Case 5 had multiple liver metastases at 11 months after surgery. This patient underwent chemotherapy with sunitinib and complete remission was achieved.

### Pathology

**Fig. 1:**
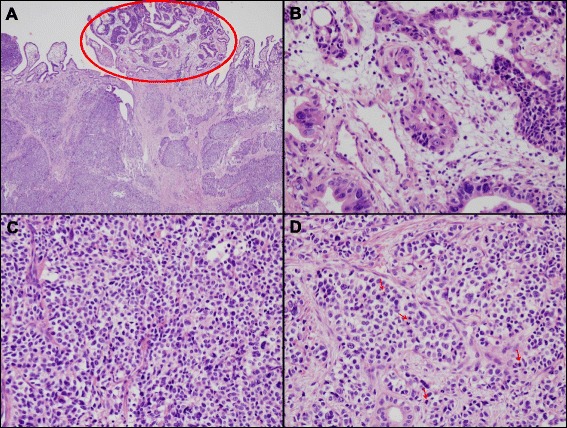
Pathologic findings of collision tumor accompanied with neuroendocrine carcinoma and adenocarcinoma. **a** *Red circle*: adenocarcinoma, well-differentiated area. Tumor cells forming gland and showing infiltrative growth. Under area: solid tumor cell nest (H&E stain, ×40). **b** High power view of adenocarcinoma area (H&E stain, ×400). Atypical epithelial cells forming gland. **c** High power view of solid nest area (H&E stain, ×400). The tumor cell showing typical “salt and pepper” chromatin pattern which is compatible with neuroendocrine tumor cells. They are positive for neuroendocrine marker, synaptophysin and chromogranin in immunohistochemistry staining. **d** The neuroendocrine tumor area showing increased mitosis (*red arrow*), and increased Ki-67 index (about 70%) (H&E stain, ×400)

All patients underwent R0 resection, which was defined as no residual tumor with negative surgical margin. The mean tumor size was 1.9 ± 0.56 cm (range, 1.2–2.0 cm). Lymph node metastases were detected in two patients. All patients were synaptophysin-positive, 2 were chromogranin-positive and 5 were CD56-positive. Lymphovascular invasion was observed in 3 patients and there was no perineural invasion in all patients. In case 2, the histological result was collision tumor accompanied with poorly differentiated neuroendocrine carcinoma at the deepest invasive portion and well differentiated adenocarcinoma at superficial portion (pT3). Neuroendocrine carcinoma accounted for 90% of the tumor mass, and adenocarcinoma 10% (Fig. 1). In this patient, liver metastases were confirmed as neuroendocrine tumor by needle biopsy 10 months after surgery. The immunohistochemical data of the 5 patients and tumor assessments according to the staging system are listed in Table 3.

**Table 3:** Histopathological and immunohistochemical data and staging

Case	Size (cm)	Lymph node metastasis (n)	Mitosis (/10HPF)	Ki-67 (%)	Lymphovascular invasion	Perineural invasion	WHO	ENETS	UICC
1	2.2	No (0/12)	1	1	Yes	No	G1	IIA	IA
2	1.2	No (0/20)	102	70	Yes	No	G3	IIB	IIA
3	1.5	Yes (1/17)	5	3	No	No	G2	IIIB	IIB
4	2.6	Yes (4/20)	4	4	Yes	No	G2	IIIB	IIB
5	2.0	No (0/50)	1	5	No	No	G2	IIA	IB

*ENETS* European Neuroendocrine Tumour Society, *UICC* International Union Against Cancer, *WHO* World Health Organization

### Survival

Median periods of follow-up were 45 months (range, 43–78 months), and complete follow-up data were available for all patients. Laboratory test and abdominal CT scan were performed every 3 months in the first 2 years after operation, and then every 6 months. Chest CT scan was performed once in a year to identify distant metastasis. 4 patients were alive at the last follow-up. Recurrence in the liver was observed in 2 patients. In these two patients, the Ki-67 index exceeded 5% and a low lymphocyte-neutrophil ratio was observed when compared to the other 3 patients (23.4% and 15.8% versus 52.7%, 40.9% and 62.1%).

## Discussion

The small bowel is most common site of neuroendocrine tumor occur, however, neuroendocrine tumor very rarely occurs at the ampulla of Vater [13]. Neuroendocrine tumors originating in the duodenum represent merely 4% of all carcinoid tumors [14]. In the previous study of Randle et al., the proportion of neuroendocrine tumors from duodenum and ampulla of Vater were 92% and 8%, respectively [15]. The common clinical feature is jaundice, similar to other ampullary tumors. In some cases, patients complain of non-specific gastrointestinal symptoms [2, 5, 16]. In the latter, it is difficult to detect an ampulla of Vater tumor without performing endoscopy or other imaging studies. In the present study, these tumors were detecting during gastroduodenoscopy of medical check-up in 4 of 5 patients; the remaining patient had jaundice.

The diagnostic modalities for NETAoVs are same as those for ampullary adenocarcinoma. CT or MRI can reveal NETAoVs as a mural and intramural enhancing mass within the submucosal region [17]. For definite diagnosis, immunohistochemical staining is needed after tumor biopsy. Because of the finding of a submucosal tumor at the ampulla on endoscopy, the rate of preoperative histological diagnosis on endoscopic biopsy is low, ranging from 14 to 66% [2, 5, 13, 18]. In the present study, a correct diagnosis of NETAoVs without symptoms was confirmed preoperatively in 4 patients for a preoperative diagnostic accuracy of 100%; the excellent results reflected the use of EUS-guided biopsy. Therefore, EUS-guided biopsy is considered to be more useful than endoscopic biopsy alone in obtaining a preoperative accurate diagnosis [16]. However, mitosis count of biopsy specimen is an incorrect assessment at present, and requires refinement.

Previous studies reported that the incidence of lymph node metastases approaches 50%, which has led to the recommendation of pancreaticoduodenectomy as the procedure of choice for NETAoVs [5, 6, 13, 19–21]. Nodal involvement appears to be of lesser significance to long-term survival [5, 6, 16, 22, 23]. Because a more advanced stage does not predict a worse prognosis, the TNM and ENETS staging systems are limited in predicting prognosis. If lymph node metastasis is not a prognostic factor, endoscopic local resection or surgical ampullectomy might be available treatment options for selective patients with NETAoVs [2, 24, 25]. Although less radical operation may decrease postoperative complication rate and preserve pancreatic function, it has the risk of incomplete removal of metastatic lymph nodes [26]. Therefore, ampullectomy can be considered for the patients with well differentiated, slow-growing and small sized tumors, who cannot be tolerable for radical operation due to high surgical risk [5].

In this series, all the patients underwent pancreaticoduodenectomy. Lymph node metastasis occurred in 2 cases; both patients remain alive without recurrence. Under the WHO classification system, our cases consisted of one neuroendocrine tumor, 3 well differentiated neuroendocrine carcinomas and one poorly differentiated neuroendocrine carcinoma. Under the TNM staging system, we had two stage I, and three stage II. Under the ENETS system, we had three stage II, and two stage III. In this study, the Ki-67 index was 5 and 70% in two cases of liver metastases. Although it is difficult to downplay the importance of the Ki-67 index in neuroendocrine tumors, further research is needed for the prognostic significance of the Ki-67 index. Low lymphocyte/neutrophil ratio is a factor reducing disease-free survival [27]. In this study, low lymphocyte/neutrophil ratio appeared to be associated with early recurrence. However, due to the limited number of patients, statistical significance was doubtful.

There is no consensus regarding adjuvant treatment for NETAoVs. We have performed adjuvant chemotherapy for the patients who had lymphovascular invasion or lymph node. As a result, adjuvant chemotherapy was performed in 4 patients in this study. One patient among them and a patient without adjuvant chemotherapy experienced recurrence.

## Conclusions

In conclusion, radical resection for NETAoVs can provide the information of status of lymph node metastasis after surgery. In present study, two of the five patients developed liver metastases within a year despite implementation of radical resection with lymph node dissection. This result suggests high aggressiveness of NETAoVs. However, correlation between lymph node metastasis and overall survival is uncertain to date due to lack of the number of NETAoVs. Regular medical check-up including gastroduodenoscopy may give a chance to detect and cure asymptomatic NETAoVs.

## Abbreviations

CT: Computed tomography
EUS: Endoscopic ultrasound
MRI: Magnetic resonance imaging
NETAoV: Neuroendocrine tumors of ampulla of Vater
